# Impact of COVID-19 on 1000 m Running and Pull-Up Performance among College Men Living in China

**DOI:** 10.3390/ijerph19169930

**Published:** 2022-08-11

**Authors:** Xiaolu Feng, Jun Qiu, Yangyang Wang, Xinyi Wen, Lili Bai, Hongjun Yu

**Affiliations:** 1 Department of Physical Education, Tsinghua University, Beijing 100084, China; 2 Institute of Sports and Health, Huzhou University, Huzhou 313000, China; 3 School of Sports Science, Tianjin Normal University, Tianjin 300387, China

**Keywords:** COVID-19, fitness testing, 1000 m running, pull-up, college students

## Abstract

Background: This study aimed to estimate the impact of the COVID-19 lockdown on fitness performance among Chinese college men during the pandemic period and to explore how fitness changed with a different college grade. Methods: We conducted repeated measures of 1000 m running and pull-up testing on students from one university in China before and after the lockdown. A total of 7107 (age 19.21 ± 1.17 yr.) male students who completed the same 1000 m running and pull-up testing in 2019 and 2020 were included in the analysis. Results: The paired t-test result indicates a reduction in 1000 m running and pull-up performance by 10.91% (95% CI = 0.89, 0.95) and 23.89% (95% CI = −0.36, −0.31), respectively. Interestingly, college men in the 2017 grade (the third-year college men) had more decreases than in the 2019 grade (the first-year college men). The 1000 m running performance was decreased by 14.43% and 6.48% in the third- and the first-year college men, respectively. The pull-up performance was decreased by 39.11 % in the third-year college men while increased by 10.98% in the first-year college men. Conclusions: The COVID-19 lockdown reduced 1000 m running and pull-up performances among Chinese college men. The reduction varies by grade and it seems to be particularly seriously decreased for the third-year college men while being modest for the first-year college men. Public policy was urgently needed to improve Chinese college men’s fitness performance after the lockdown.

## 1. Introduction

On 11 March 2020, the respiratory disease caused by COVID-19 was declared a pandemic by the World Health Organization (WHO) and a national emergency in China. To date (29 June 2022), 4,249,438 confirmed cases of COVID-19 with 21,073 deaths were reported to the National Health Commission of the People’s Republic of China [1]. To prevent the spread of COVID-19, the Chinese government enacted numerous restrictions on human movement and physical interactions. From mid-January 2020, all schools and universities closed in all provinces until mid-June 2020 [2,3]. As a result, college students stayed at home and no longer had access to college-based physical activities such as physical education, recess, and exercise. Outdoor physical education classes for college students, such as track and field, martial arts, basketball, football, volleyball, tennis, etc., were canceled and replaced with web-based courses until early June or later. Simultaneously, national, provincial, and local public parks, playgrounds, and sports venues were closed to prevent crowds from gathering in China.

To slow the spread of COVID-19, school closure and home confinement measures were necessary. However, they also created various effects in different domains, such as physical inactivity, weight gain, irritability, depression, and anxiety [4,5,6,7,8]. As rapid physical activity (PA) reduction has been a worldwide tendency during the pandemic [9], adolescents’ physical fitness is tended to be negatively affected. According to a survey of 264 teenage students in the United States, both male and female students showed a significant reduction in physical fitness performance during the COVID-19 pandemic [10]. Meanwhile, previous studies have observed the reduction of physical fitness in professional athletes [11,12,13]. For instance, Ambrozy et al., [13] evaluated 20 international level kickboxers’ physical fitness before the COVID-19 outbreak and during the pandemic after reopening sports facilities. They found that the temporary closing of sports clubs had significantly deteriorated physical fitness.

To prevent the adverse effects of COVID-19 on physical fitness among adolescents, many schools and colleges forced students to participate in web-based physical education (PE) classes at home instead of on the playground [14]. Although learning at home has given students more leisure time for physical activity, there might be enormous disparities in access to these opportunities based on socioeconomic status, neighborhood characteristics, partners, and friends from home [15,16]. However, few studies [17] estimated the effectiveness of online physical education classes for holding back the adverse fitness effects of COVID-19.

Furthermore, reducing PA levels and physical fitness during the COVID-19 pandemic would probably cause health problems for college students. Globally, nearly 80% of adolescents are insufficiently active [18,19]. It is likely to contribute to critical global health problems, including cardiovascular diseases risk [20,21] and mental health disorders [22]. In China, more than 77% of students at school failed to meet the WHO PA recommendation guideline [23,24,25] and the COVID-19 pandemic would lead to reduced PA that adversely affected students’ fitness [26,27,28]. Moreover, a previous study suggested that school closure during the COVID-19 lockdown would lead to increased risk of obesity and decreased sports performance in college students [17].

Despite the previous work, two main gaps exist in the earlier studies. First, although previous studies have stated the decline of PA levels during the COVID-19 pandemic, which might lead to the potential negative impact on fitness [9,29,30], few studies reported the kind of impact of the COVID-19 pandemic on human fitness. Second, most studies examined the effect of the COVID-19 lockdown on fitness based on small samples of hundreds [10,31] or self-reported physical activity levels [32,33], but, to our best knowledge, few studies estimated the effect based on objective fitness testing with large samples.

To address this knowledge gap, this study aims to estimate the effects of the COVID-19 pandemic on Chinese college men’s fitness performance (1000 m running and pull-ups). Furthermore, this study also intends to investigate potential differences between college men of different grades. In this case, the novelty of this study was performing repeated tests on a large sample of 7107 people in 1000 m running and pull-up performances pre- and post-COVID-19. This study would provide data to contrast the effects of the COVID-19 pandemic on physical fitness in a large sample among college men in China and make a classification of the subjects by grade. It would provide novel insight into the effects of COVID-19 on the physiological states of the population.

## 2. Materials and Methods

### 2.1. Participants

A total of 7107 college men completed the physical fitness testing in 2019 (pre-COVID-19 lockdown) and 2020 (post-COVID-19 lockdown). The college men were from three different grades: 2171 college men were from the 2017 grade (college men who entered college in 2017); 2437 college men were from the 2018 grade (college men who entered college in 2018); 2500 college men were from the 2019 grade (college men who entered college in 2019). Participants who completed the 1000 m running and pull-up performance tests in 2019 and 2020 were included in the analysis of this study.

### 2.2. Procedures

At Tsinghua University, China, all college students must participate in physical fitness testing every autumn semester with exceptions for special conditions, such as illness or disability. According to the National Student Physical Health Standard (NSPHS) in China [34], the physical fitness testing for college students includes height and weight testing for both college men and college women, while pull-ups and 1000 m running for college men only. For college men, 1000 m running and pull-up performances were the main measurement indicators for assessing their cardiorespiratory fitness [35] and muscular strength [36,37].

Trained teams took the 1000 m running and pull-up testing pre- and post-COVID-19 lockdown. The period of the COVID-19 lockdown for Tsinghua University students was from mid-Jan 2020 to late-August 2020. During the lockdown period, all students stayed at home and no longer had access to college-based physical activities. After late August, the college students returned to school and engaged in college-based physical activities. In this context, the pre-COVID-19 testing was performed from 9 September to 10 November 2019, and the post-COVID-19 testing was performed from 19 October to 14 November 2020. The testing was carried out from 8:00 am to 12:15 am and 1:30 pm to 6:40 pm. The testing procedures and measurements were the same in the pre- and post-COVID-19 testing. None of the participants tested positive for COVID-19 before the post-testing. Tsinghua University ethics review boards approved the study (IRB #201253400). Participants provided written informed consent before the testing.

### 2.3. Measurement

#### 2.3.1. 1000 m Running

The 1000 m running testing was conducted on the playground of Tsinghua University and measured by the time of a 1000 m race recorded in seconds. Before the 1000 m running testing, participants were asked to warm up under the guidance of our trained teams. During the testing, participants were asked to wear a vest containing a timing chip. The tester could automatically measure participants’ running time using an infrared sensor. Participants were asked to finish the 1000 m running as fast as possible, and their running time in the range of 00:00–16:66 (min:sec) was recorded and included in this study. Each participant only performed the 1000 m running testing once. The tester used the running tester of Tongfang Health Fitness Testing Products 5000 series (Tongfang Health Technology Co., Ltd., Beijing, China) [38]. The tester could automatically measure the running time (range: 0–999.9 s, resolving: 0.1 s, precision: ±0.5%) with an infrared sensor and anti-jamming design.

#### 2.3.2. Pull-Ups

The pull-up testing was conducted in the fitness square of Tsinghua University. Before the pull-up testing, participants were asked to warm up under the guidance of our trained teams. After warming up, we measured the pull-up testing by the repetitions of the completed required action. Participants need to pull themselves up until their chin clears the bar with an overhand grasp and finish by lowering the body until they fully extended their arms and shoulders. The number of repetitions they performed the pull-up action until they were fully exhausted were be recorded. Each participant performed the pull-up testing one time. Their actions in the range of 0–100 repetitions were recorded and included in this study. The tester used was the pull-up tester of Tongfang Health Fitness Testing Products 5000 series (Tongfang Health Technology Co., Ltd., Beijing, China) [39]. The tester could measure the number of participants’ pull-up repetitions (range: 0–100 repetitions, resolving: 1 time, precision: ±1 time) with a smart counting design and the testing data could transfer to a personal computer automatically.

### 2.4. Statistical Analyses

To sum up and compare the characteristics of the overall sample, descriptive statistics of this research used means and standard deviations and percentages. One-way ANOVA was used for the comparison of the baseline characteristics of 2017, 2018, and 2019 grade college men. We analyzed participants’ physical fitness level changes before and after the lockdown comparison. We performed a two-way ANOVA to examine the groups and time as factors to assess the interaction between groups and time. A paired *t*-test was conducted to compare mean differences in the same sample before and after the COVID-19 lockdown. In the analysis, participants’ 1000 m running and pull-up performances before and after the lockdown were performed by paired t-test analysis. We performed paired *t*-test using bootstrapping and stratified age and BMI variables as covariates. In addition, we analyzed the effect size of Cohen’s d (small effect size, 0.2 ≤ d < 0.5; medium effect size, 0.5 ≤ d < 0.8; large effect size, d ≥ 0.8) and compared the differences among the measurement points in the 2017 grade college men, the 2018 grade college men, and the 2019 grade college men. The fitness changes compared with the 2019 baseline as the reference. For example,

(1) $$2020\;fitness\;change\;\%=\frac{fitness\;test\;in\;the\;2020\;year-fitness\;test\;in\;the\;2019\;year}{fitness\;test\;in\;the\;2019\;year}\times 100\%.$$

We also analyzed a subgroup of the fitness change differences among the 2017 grade men, 2018 grade men, and 2019 grade men. We performed all the statistical tests using IBM SPSS Statistics 28.0 (SPSS, Inc., Chicago, IL, USA). The level of statistical significance was set at *p* < 0.05.

## 3. Results

### 3.1. The Characteristics of Participants

Table 1 summarizes the baseline characteristics of the sample in the 2019 year. A total of 7107 participants were composed of college men at Tsinghua University. The participants from 2017, 2018, and 2019 grades were 30.54%, 34.29%, and 35.17%, respectively. The mean age of participants was 19.21 ± 1.17 years and the mean age of participants from 2017, 2018, and 2019 grades was 20.17 ± 0.82 (years), 19.17 ± 0.75 (years), and 18.23 ± 0.82 (years), respectively. The mean BMI was 22.39 ± 3.59 (kg/m^2^) and the mean BMI of participants from 2017, 2018, and 2019 grade was 22.90 ± 3.62 (kg/m^2^), 22.31 ± 3.58 (kg/m^2^) and 21.94 ± 3.46 (kg/m^2^), respectively. The mean level of 1000 m running performance of all the participants was 4:05 ± 0:28 (min:sec) and the mean level of 1000 m running performance of participants from 2017, 2018, and 2019 grades was 4:13 ± 0:29 (min:sec), 4:03 ± 0:26 (min:sec), and 4:00 ± 0:27 (min:sec), respectively. The mean level of pull-up performance of all the participants was 7.42 ± 7.21 (repetitions) and the mean level of pull-up performance of participants from 2017, 2018, and 2019 grades was 9.04 ± 7.30 (repetitions), 9.06 ± 7.59 (repetitions), and 4.35 ± 5.57 (repetitions), respectively.

### 3.2. The Physical Fitness Testing Result

Table 2 presents the descriptive statistics of the physical fitness testing results of participants in 2019 (pre-COVID-19 lockdown) and 2020 (post-COVID-19 lockdown). Based on the significant differences in the baseline of college men in different grades, we also performed a two-way ANOVA to examine the groups and time as factors to assess the interaction between groups (2017, 2018, and 2019 grades) and time (pre-COVID-19 testing and post-COVID-19 testing). The results showed that the 1000 m running mean time of participants in the 2019 and 2020 years were 4:13 ± 0:26 vs. 4:49 ± 0:44 (min:sec), 4:02 ± 0:24 vs. 4:32 ± 0:32 (min:sec), and 3:59 ± 0:25 vs. 4:14 ± 0:28 (min:sec) for the 2017, 2018, and 2019 grade men, respectively. The pull-up mean repetitions of participants in the 2019 and 2020 years were 9.12 ± 7.06 vs. 5.56 ± 5.66 (repetitions), 9.29 ± 7.58 vs. 6.66 ± 7.80 (repetitions), and 4.35 ± 5.51 vs. 4.83 ± 5.56 (repetitions) for the 2017, 2018, and 2019 grade men, respectively. The differences among college men in the 2017, 2018, and 2019 grades were statistically significant (*p* < 0.001) in the 1000 m running performance (F = 1158.49, *p* < 0.001) and the pull-up performance (F = 294.02, *p* < 0.001), pre- and post-COVID-19 lockdown.

### 3.3. Impact of COVID-19 on Fitness Performance

Table 3 reports the paired t-test outcomes for physical fitness testing before and after the COVID-19 lockdown. The results showed that the participants’ 1000 m running and pull-up performances significantly declined before and after the lockdown (*p* < 0.001).

The participants’ 1000 m running level decreased by 10.91% in total (*p* < 0.001) and the effect size was large (d = 0.92). Furthermore, the 1000 m running level decreased for 2017, 2018, and 2019 grade men by 14.43% (*p* < 0.001), 12.09% (*p* < 0.001), and 6.48% (*p* < 0.001), respectively. The effect size for the 2017 and 2018 grades was large (d = 1.03, d = 1.31, respectively) and the effect size for the 2019 grade was medium (d = 0.73) (see Table 3).

The participants’ pull-up levels decreased by 23.89% in total (*p* < 0.001) and the effect size was small (d = 0.33). In addition, the pull-up levels decreased for the 2017 and 2018 grade men by 39.11% and 28.31%, respectively. The effect size for the 2017 and 2018 grades was medium (d = 0.78) and small (d = 0.39). Interestingly, the results show that the 2019 grade men’s pull-up levels increased by 10.98% and the effect size was small (d = 0.14) (see Table 3).

## 4. Discussion

This study assessed the impact of the COVID-19 lockdown on Chinese college men’s 1000 m running and pull-up performances by grade. We found that Chinese college men significantly decreased their 1000 m running and pull-up performances due to the COVID-19 lockdown. Furthermore, we found that participants’ fitness was differently decreased by grade. Compared with the 2019 grade men, it seemed to be a more serious decrease for the 2017 and 2018 grade men. Interestingly, we also found that the 2019 grade men’s pull-up testing results performed better after the school closure. To the best of our knowledge, this is the first finding that examines the impacts of the COVID-19 lockdown on 1000 m running and pull-up performances for Chinese college men by grade.

In this study, we provided evidence to confirm the negative impact of the COVID-19 lockdown on the 1000 m running and pull-up performances among college men living in China. In our study, we found that college men’s 1000 m running and pull-up levels totally significantly decreased by 10.91% (*p* < 0.001) and 23.89% (*p* < 0.001), respectively. This finding was consistent with a previous study showing that adolescents decreased their fitness levels after the COVID-19 lockdown [13,40,41]. Previous research reported that the increasing physical inactivity and sedentary behavior during the COVID-19 pandemic might increase the decline of physical fitness [41]. Ambrozy et al., reported that the participants’ mean level of 1000 m running performance decreased by 3.84% in total and pull-up performance levels decreased by 12.69% after two months of lockdown in Poland, respectively [13]. Similarly, China’s lockdown lasted five months and college men living in China also showed a declining trend in their physical fitness. However, they experienced a more significant decline in 1000 m running (decreased by 10.91% in total) and pull-up (decreased by 23.89% in total) performances during the longer lockdown.

Moreover, this finding was inconsistent with a recent study revealing that college men’s pull-up performances increased during the lockdown [17]. This study indicated that pull-up performances increased by 25% among college students in Wuhan, China after social isolation [17]. Since the universities in this study and our study were both in China, participants’ duration of lockdown in the two studies was almost the same. A possible explanation for this difference could be the different dates we carried out the testing. Xia et al., carried out the fitness testing at the beginning of the semester, while our study was in the middle of the semester (from 9 September to 10 November 2019 and from 19 October to 14 November 2020). PE courses and college men’s strength exercise behavior might affect Tsinghua university. Our study found that college men’s pull-up level was higher than in the previous study (seven repetitions vs. four repetitions). Even after the lockdown, our participants’ mean level of pull-up (5.69 repetitions) was higher than the participants in the previous study (five repetitions).

Interestingly, our study found that the impact of COVID-19 on physical fitness among college men varies by grade. Although the 2017 and 2018 grade men significantly declined on the 1000 m running and pull-up performances, the 2019 grade men increased their pull-up performance by 10.98%. A possible explanation for this finding was PE courses could play a crucial role in maintaining and improving college students’ pull-up performances. In our study, before the COVID-19 lockdown, the college men’s pull-up mean levels of 2017, 2018, and 2019 grades was 9.12, 9.29, and 4.35 repetitions, respectively. Thus, we could infer that before the COVID-19 lockdown, college men in 2017 and 2018 grades had experienced more than one year’s campus PE training, while college men in 2019 grades just experienced a short period of campus PE training. Our participants might have a stronger fitness in college before the lockdown and are more likely to be affected by the school closure.

Several factors could contribute to the decline of college men’s physical fitness. First, a possible reason for the decrease in college men’s physical fitness was that indoor or outdoor PE courses had been replaced by web-based PE courses [17]. Due to the lockdown, web-based PE courses might decrease exercise volume and intensity. Second, during the COVID-19 lockdown, school closure and home confinement measures could disregard college men’s physical activity behavior. A previous study has proved that the COVID-19 lockdown had led to a reduction in physical activity among Chinese university students and physical activity decreased more for male than female students [42]. Moreover, previous research reported that physical activity behavior was linked with physical fitness [41,43]. Therefore, the decline of college men’s physical fitness level could be associated with PA reduction during the COVID-19 lockdown, which had been reported [9,22,32]. In this case, it is urgent to intervene in promoting physical activity for Chinese college men. Colleges should take more measures to improve college men’s fitness (such as offering more recreation facilities) and encourage them to do more physical activity and to avoid inactivity and other sedentary behavior. The government should formulate policies to open more public parks, playgrounds, and sports venues to college students. Furthermore, the government should reduce the COVID-19 lockdown to avoid these negative effects. In addition, considering that COVID-19 is not completely gone, a repeat of the COVID-19 lockdown remains a possibility. It is also necessary to strengthen family-based physical activity and exercise for college men.

### Strength and Limitations

The strength of our study is that this is a repeating test on the same sample and a large sample in 1000 m running and pull-up levels of Chinese college men by grade before and after the COVID-19 lockdown. Nevertheless, our study also has limitations. Firstly, although college men’s cardiorespiratory fitness and muscular strength are usually assessed by 1000 m running and pull-up performances in China, the 1.5 mile and 12 min walk/run tests, which represent useful alternatives for estimating cardiorespiratory fitness [44], should be used to assess health-related physical fitness in further studies. Secondly, this study made a classification of the subjects by grade and only men were included. Future studies could analyze BMI, gender, and other levels of physical condition for more interesting findings. Thirdly, this study assessed the 1000 m running and pull-up levels of a sample of college men from only one Chinese university. Therefore, the findings cannot be extended to the whole population of college men in China. Further studies need to consider the replication of our findings in other universities.

## 5. Conclusions

COVID-19 decreased Chinese college men’s 1000 m running and pull-up performances. The impact varied by grade and it seemed particularly serious for the third-year college men while being modest for the first-year college men. Public policy was urgently needed to improve Chinese college men’s fitness performance after the lockdown.

## Figures and Tables

**Table 1:** The baseline characteristics of the sample in the 2019 year.

College Men	2017 Grade	2018 Grade	2019 Grade	Total	*p*
N	2171 (30.54%)	2436 (34.29%)	2500 (35.17%)	7107 (100%)	
Age (year), mean (SD)	20.17 (0.82)	19.17 (0.75)	18.23 (0.82)	19.21 (1.17)	<0.001
1000 m running (min:sec), mean (SD)	4:13 (0:29)	4:03 (0:26)	4:00 (0:27)	4:05 (0:28)	<0.001
Pull-up (repetitions), mean (SD)	9.04 (7.30)	9.06 (7.59)	4.35 (5.57)	7.42 (7.21)	<0.001

**Table 2:** College men’s physical fitness performance differences pre- and post-COVID-19 lockdown by grade.

College Men	2017 Grade	2018 Grade	2019 Grade	F	*p*
1000 m running, mean (SD)					
N	1936	2241	2332		
2019 year (min:sec)	4:13 (0:26)	4:02 (0:24)	3:59 (0:25)	1158.49	<0.001
2020 year (min:sec)	4:49 (0:44)	4:32 (0:32)	4:14 (0:28)		
Pull-up, mean (SD)					
N	1937	2226	2293		
2019 year (repetitions)	9.12 (7.06)	9.29 (7.58)	4.35 (5.51)	294.02	<0.001
2020 year (repetitions)	5.56 (5.66)	6.66 (7.80)	4.83 (5.56)		

**Table 3:** Paired *t*-test results of physical fitness testing in the 2019 year and the 2020 year.

Fitness Test Change (2020–2019)	Paired Difference Mean (95% CI)	Performance (%)	Effect Size (95% CI)	*t*
1000 m running (s)				
2017 grade	36.43 (34.84, 38.02)	14.43↓	1.03 (0.97, 1.08)	44.81 ***
2018 grade	29.29 (28.35, 30.22)	12.09↓	1.31 (1.25, 1.37)	61.51 ***
2019 grade	15.47 (14.59, 16.35)	6.48↓	0.73 (0.68, 0.77)	34.65 ***
Total	26.64 (25.93, 27.34)	10.91↓	0.92 (0.89, 0.95)	74.00 ***
Pull-up (repetitions)				
2017 grade	−3.57 (−3.78, −3.36)	39.11↓	−0.78 (–0.83, –0.73)	−33.52 ***
2018 grade	−2.63 (−2.91, −2.35)	28.31↓	−0.39 (−0.44, –0.35)	−18.24 ***
2019 grade	0.48(0.34, 0.62)	10.98↑	0.14 (0.10, 0.18)	6.77 ***
Total	−1.78 (−1.65, −1.92)	23.89↓	−0.33 (−0.36, −0.31)	−26.53 ***

Notes: *** *p* < 0.001; ↑ increase; ↓ decrease.

## Data Availability

The datasets generated and/or analyzed during the current study are not publicly available due to confidentially reasons but are available from the corresponding author on reasonable request.

## Abbreviations

PA—physical activity; PE—physical education; COVID-19—Corona Virus Disease 2019; NHCPRC—National Health Commission of the People’s Republic of China; MOEPRC—Ministry of Education of the People’s Republic of China.
